# Changes in autonomy, job demands and working hours after diagnosis of chronic disease: a comparison of employed and self-employed older persons using the English Longitudinal Study of Ageing (ELSA)

**DOI:** 10.1136/jech-2017-210328

**Published:** 2018-06-23

**Authors:** Maria Fleischmann, Ewan Carr, Baowen Xue, Paola Zaninotto, Stephen A Stansfeld, Mai Stafford, Jenny Head

**Affiliations:** 1 Department of Epidemiology and Public Health, University College London, London, UK; 2 Institute of Psychiatry, King’s College London, London, UK; 3 Centre for Psychiatry, Wolfson Institute of Preventive Medicine, Barts and the London School of Medicine and Dentistry, Queen Mary University of London, London, UK; 4 MRC Unit for Lifelong Health and Ageing, University College London, London, UK

**Keywords:** workplace, chronic disease, gerontology, longitudinal studies

## Abstract

**Background:**

Modifications in working conditions can accommodate changing needs of chronically ill persons. The self-employed may have more possibilities than employees to modify their working conditions. We investigate how working conditions change following diagnosis of chronic disease for employed and self-employed older persons.

**Methods:**

We used waves 2–7 from the English Longitudinal Study of Ageing (ELSA). We included 1389 participants aged 50–60 years who reported no chronic disease at baseline. Using fixed-effects linear regression analysis, we investigated how autonomy, physical and psychosocial job demands and working hours changed following diagnosis of chronic disease.

**Results:**

For employees, on diagnosis of chronic disease autonomy marginally decreased (−0.10, 95% CI −0.20 to 0.00) and physical job demands significantly increased (0.13, 95% CI 0.01 to 0.25), whereas for the self-employed autonomy did not significantly change and physical job demands decreased on diagnosis of chronic disease (−0.36, 95% CI −0.64 to –0.07), compared with prediagnosis levels. Psychosocial job demands did not change on diagnosis of chronic disease for employees or the self-employed. Working hours did not change for employees, but dropped for self-employed (although non-significantly) by about 2.8 hours on diagnosis of chronic disease (−2.78, 95% CI −6.03 to 0.48).

**Conclusion:**

Improvements in working conditions after diagnosis of chronic disease were restricted to the self-employed. This could suggest that workplace adjustments are necessary after diagnosis of chronic disease, but that the self-employed are more likely to realise these. Policy seeking to extend working life should consider work(place) adjustments for chronically ill workers as a means to prevent early exit from work.

## Introduction

A growing share of the population in developed countries is diagnosed with one or more chronic diseases such as diabetes, hypertension, arthritis, cancer or coronary heart disease. In the European Union (28 countries), about 45% of men and women aged 55–64 years had a long-standing illness or health problem in 2015.^1^ People with a chronic disease are at increased risk of not being in employment.^2^ The average employment rate of people with two or more chronic diseases was 50%, compared with 74% for those without chronic disease.^3^

Good working conditions may maximise older persons’ work participation. Research has shown that good working conditions, such as high work time control,^4^ high job control^5^ and decision authority,^6^ were associated with later retirement, but that poor working conditions, for example, low skill discretion and high conflict at work,^7^ job pressure,^8 9^ decreased decision latitude and increased job demands,^10^ high job demands and low recognition,^6^ were related to earlier (intended) retirement and higher risk of sickness absence. As expected, workers with chronic disease had lower work performance, reported more absenteeism and were more likely to leave work early, compared with workers without chronic disease.^11–15^ Good working conditions may mitigate the negative effects chronic disease has for work participation.^15^ Among persons with chronic disease, higher job autonomy was associated with reduced risk of sickness absence and disability benefit.^16 17^ Conversely, low psychosocial resources were linked to increased risk of work exit for chronically ill persons.^14^

Considering that working conditions are modifiable aspects of the job,^6 18^ Ilmarinen argues that it is of the utmost importance for occupational health not only to ‘treat’ chronically ill workers, but also improve their working conditions.^19^ Older persons with chronic disease may have different work requirements and lower work capability, compared with workers in good health.^14 20^ Many older persons work for employers and require approval when it comes to adjusting working conditions to their changing needs. This stands in sharp contrast to the self-employed, who can more autonomously modify their working conditions.^21 22^ In line with this, prior research reports higher job control among self-employed than among employed workers.^23^ In this study, we presume that working conditions are modifiable aspects of the job that might be adapted to accommodate changing needs. By comparing employees to the self-employed, we contrast two groups that differ with respect to whether modifications can be made autonomously. Specifically, we investigate how diagnosis of chronic disease affects autonomy, physical job demands, psychosocial job demands and working hours of employed and self-employed older persons.

## Methods

We used data from the English Longitudinal Study of Ageing (ELSA), a national representative study of people aged ≥50 years living in private households in England. Details of the study are given elsewhere.^24^ The first interviews in 2002/2003 included a representative sample of 11 392 participants (core sample). Respondents were interviewed biennially and new refresher study members were added at wave 3 (2006/2007) and wave 4 (2008/2009).

### Sample selection

**Figure 1:**
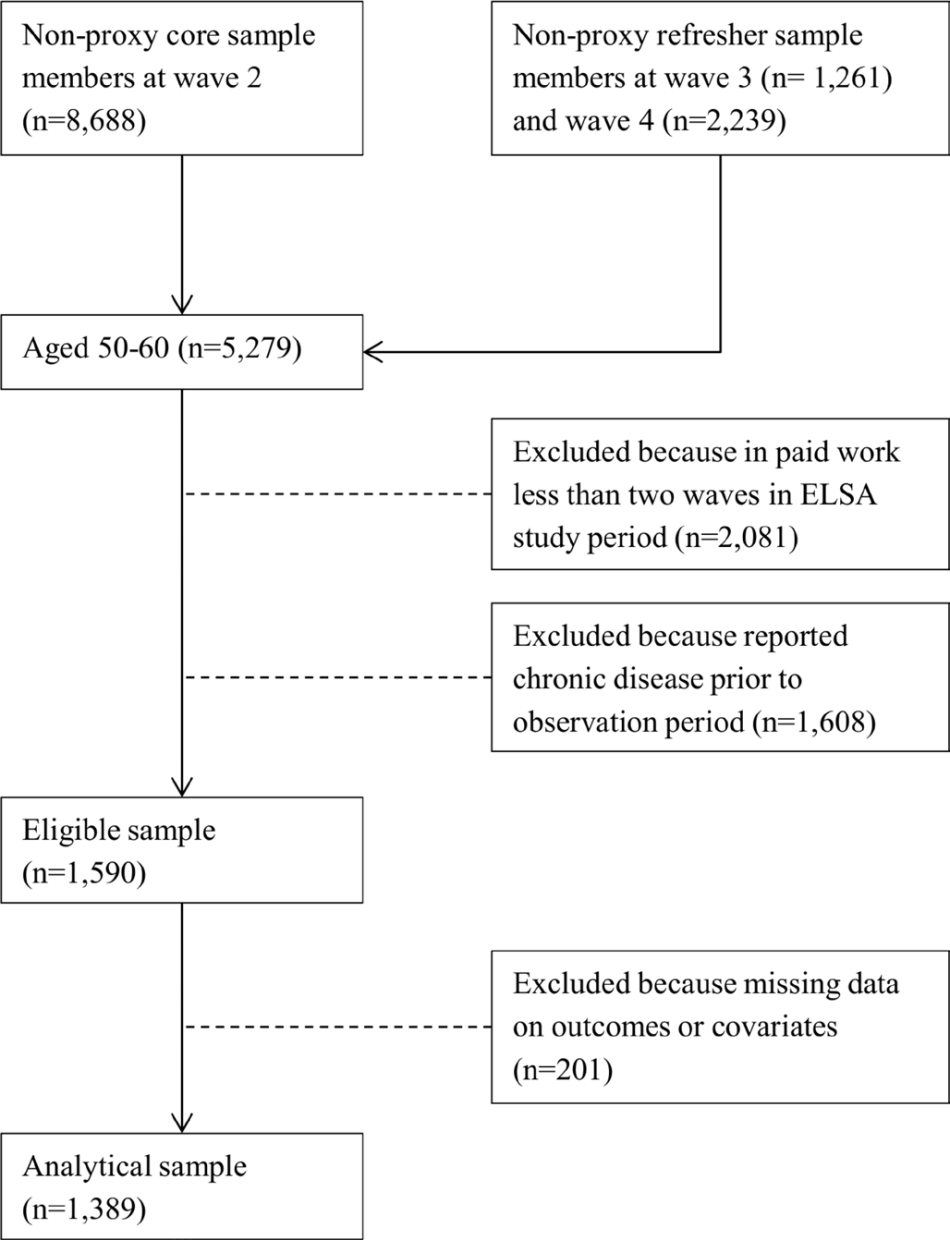
Flow chart sample selection. ELSA, English Longitudinal Study of Ageing.

Our baseline sample consists of the non-proxy core sample members at wave 2 (n=8688), and the non-proxy refresher samples at wave 3 (n=1261) and wave 4 (n=2239). Proxy interviews (about 2% of all interviews) were pursued if eligible respondents were temporarily impaired or refused, but appointed someone to do the interview on their behalf.^25^ We did not use the first wave of ELSA (2002/2003) because information on autonomy, physical and psychosocial job demands was not available. The analytical sample (see figure 1) consisted of respondents aged 50–60 years, who were in paid work on two or more occasions and who reported no chronic disease when entering the study. We decided to focus on this age range because it represents the period in which many older persons become chronically ill, but are still in work. Including younger respondents might result in an over-representation of ‘extreme’ cases and bias our results.

We excluded respondents with missing data on outcomes or covariates. Our analyses rely on 4824 observations nested in 1389 respondents. Respondents were observed 3.5 times on average. We used data on diagnosis of chronic disease and working conditions between wave 2 (2004–2005) and wave 7 (2013–2014).

### Working conditions

Working conditions were time varying and measured in each wave 2–7 by several items derived from the Job Content Questionnaire.^26^ *Job autonomy* was based on answers to the statement ‘I have very little freedom to decide how I do my work’ (‘strongly agree’, ‘agree’, ‘disagree’ or ‘strongly disagree’). Higher values on the 4-point scale indicate higher job autonomy. *Physical job demands* were operationalised as a sum scale from two items; the physical effort required in the job (‘sedentary occupation’, ‘standing occupation’, ‘physical work’ or ‘heavy manual work’) and answers to the statement ‘My job is physically demanding’ (‘strongly agree’, ‘agree’, ‘disagree’ or ‘strongly disagree’). The sum scale ranged from two to eight. *Psychosocial job demands* were the sum of the answers to the statements ‘Considering the things I have to do at work, I have to work very fast’ and ‘I am under constant pressure due to a heavy workload’ (‘strongly agree’, ‘agree’, ‘disagree’ or ‘strongly disagree’). Again, answers ranged from two to eight. Where necessary, items were reverse coded so that higher values indicate higher physical or psychosocial job demands. *Working hours* were measured as the sum of weekly working hours in the main job and, if applicable, the second job (around 8% of the sample). Working hours excluded meal breaks and included paid overtime. Answers above 80 working hours per week were capped at 80 hours (<1% of the sample).

### Chronic disease

At each interview, respondents were asked whether they had been diagnosed by a doctor with any of the following conditions: chronic lung disease (such as chronic bronchitis or emphysema); asthma; arthritis (including osteoarthritis or rheumatism); cancer or malignant tumour (excluding minor skin cancers); high blood pressure or hypertension; diabetes or high blood sugar; stroke (cerebral vascular disease); and heart problem, that is, angina, heart attack (including myocardial infarction or coronary thrombosis), congestive heart failure, heart murmur, abnormal heart rhythm, valvular heart disease, ischaemic heart disease (including bypass grafts) and any other heart trouble. From information at each wave (2–7), we generated a time-varying variable that was coded 0 if respondents had not been diagnosed with any of the chronic conditions at that wave and 1 if they reported one or more chronic conditions.

### Self-employment

Self-employment was a time-varying indicator measuring whether individuals described their current situation as self-employed, rather than employed, in the respective wave. This variable is a derived variable available in the dataset identifying self-employed respondents based on several employment questions, including ‘are you self-employed in your main job’ and ‘are you working for yourself’.^27^

### Covariates

We include respondents’ *age* in categories at each wave (age 50–54, 55–59, 60–64, 65 and over). *Depressive symptoms* were included as a covariate, because poor mental health might affect reporting of working conditions and could be related to incidence of chronic illness. Depressive symptoms are measured at each wave based on the short form of the Centre for Epidemiologic Studies Depression (CES-D) scale (eight items).^28^ Higher values on the CES-D scale refer to more depressive symptoms, that is, worse mental health. We adjust for individuals’ *job autonomy* at each wave when analysing job demands and working hours. We also adjust for *wave*.

### Statistical analyses

We estimate linear fixed effects regression models. In fixed effects models, the interest lies solely in explaining within-person variation. Only changes within persons are considered and all time-invariant covariates (eg, gender and educational degree), even if they are unobserved, are accounted for.^29 30^ Accounting for unobserved heterogeneity allows us to be more certain that our results depict a causal impact of diagnosis of chronic disease for older persons’ working conditions. In the analyses, respondents serve as their own reference, that is, they are compared with themselves at several time points. Effects can be interpreted as average changes within individuals on the outcome variable. In our study, this approach is advantageous because self-reported information on working conditions could be biased by individuals’ perception, but would be similarly biased over time. As individuals are compared with themselves, such a bias would hence not affect our results.

We consider diagnosis of first chronic disease. Respondents without chronic disease are included in the analyses as a control group to account for temporal heterogeneity, that is, changes to the outcome over time.^31^ This allows us to estimate a time trend of how working conditions change over time. To estimate the impact of diagnosis of chronic disease and to assess the time trend since diagnosis, we included dummy variables. These represent (with a value of 1) the wave at diagnosis of chronic disease, and the first (T+1) or second (T+2) wave after diagnosis. The reference category (value 0) refers to waves without chronic disease. Later waves after diagnosis are censored due to small sample size. Additionally, we test whether the impact of diagnosis and the time trend after diagnosis differ for employed and self-employed older persons. To do so, we include interactions between the indicators of chronic disease and self-employment. All analyses were conducted in Stata V.15 (command *xtreg*) and adjusted for covariates described above. We estimated clustered sandwich estimators to account for the clustering of study phases within respondents. Results are depicted graphically.

### Sensitivity analyses

First, if working conditions are more easily modifiable in self-employment than employment, moving to self-employment could be a way to remain working despite worsening health. In additional analyses, we restrict our sample to those who are either employed throughout or self-employed throughout, that is, we excluded those who moved either from employment to self-employment (n=96) or vice versa (n=31), or had several of these moves (n=16). Second, we adjusted for whether respondents had onset of second chronic disease. Next to the variables measuring diagnosis of chronic disease described above, we included a time-varying indicator that is coded 1 in case someone was diagnosed with a second chronic disease, and 0 otherwise. Finally, we account for the possibility of reverse causation by including a dummy variable, labelled ‘anticipation effect’, which was coded 1 in the wave before diagnosis of the chronic disease and 0 otherwise.^29^ Significant results for this variable would provide some indication that the direction of the effect under investigation is reversed, that is, poor working conditions triggered chronic disease. Based on Karasek’s demand-control model, stating that high job strain—the combination of high demands and low control—related to poor mental and physical health,^18^ prior research showed that, for example, high job strain and long working hours were associated with multiple physical health symptoms.^32–35^

## Results

The analytical sample included 1389 participants, 50% of which are women (table 1). Respondents were on average aged 54.7 years when first observed. About 43% of the sample has a socioeconomic position in a ‘managerial or professional occupation’, 25% in ‘intermediate occupations’ and about 29% work in ‘routine and manual occupations’. In total, 510 of the 1389 sample members were newly diagnosed with a chronic illness while under observation. Mean age at diagnosis was about 60 years. Most respondents (73.5%) reported one chronic disease in the observation period; the remaining reported two or more chronic diseases. Of the first chronic disease reported, the most common was hypertension (32.7%), followed by arthritis (28.4%). Several respondents (10.6%) reported diagnosis of two or more chronic illnesses at the same time, as their first illness.

**Table 1:** Descriptive information, based on analysis sample

	Percentage (n)	Mean	SD
**Background information included in analyses**			
Age categorical, years (n=4824)			
45–54	21.3 (1027)		
55–59	48.9 (2360)		
60–64	25.8 (1244)		
65+	4.0 (193)		
Wave (n=4824)			
2	13.6 (656)		
3	19.1 (920)		
4	21.3 (1026)		
5	19.8 (953)		
6	15.3 (736)		
7	11.0 (533)		
Time-varying indicators (n=4824; n=1389)*			
Physical job demands		4.3	(1.7; 0.7)
Psychosocial job demands		5.0	(1.5; 0.9)
Working hours		34.9	(13.6; 6.7)
Depressive symptoms (GHQ Scale)		0.9	(1.5; 1.0)
Job autonomy		3.0	(0.8; 0.5)
**Background information not included in analyses** **(time constant) (n=1389)**			
Diagnosis chronic disease	36.7 (510)		
Age at diagnosis		59.5	(3.9)
Number of chronic diseases			
1	73.5 (375)		
≥2	26.5 (135)		
First diagnosis chronic disease			
Asthma	3.5 (18)		
Arthritis	28.4 (145)		
Cancer or malignant tumour	8.8 (45)		
Chronic lung disease	3.1 (16)		
Diabetes	3.7 (19)		
Heart problem	8.2 (42)		
Hypertension	32.7 (167)		
Stroke	0.8 (4)		
Combination of two	10.6 (54)		
Gender			
Male	50.3 (699)		
Female	49.7 (690)		
Education (ISCED)			
No qualification, NA	16.2 (225)		
Upper secondary	32.1 (446)		
Postsecondary non-tertiary	14.5 (201)		
First stage of tertiary	22.3 (310)		
Do not know/missing/other	14.9 (207)		
NS-SEC			
Managerial and professional occupations	43.3 (602)		
Intermediate occupations	24.8 (345)		
Routine and manual occupations	29.3 (407)		
Missing	2.5 (35)		

Note, *For time-varying indicators, overall SD and within SD reported.

GHQ, General Health Questionnaire; ISCED, International Standard Classification of Education; NS-SEC, National Statistics Socioeconomic Classification, three classes.

**Figure 2:**
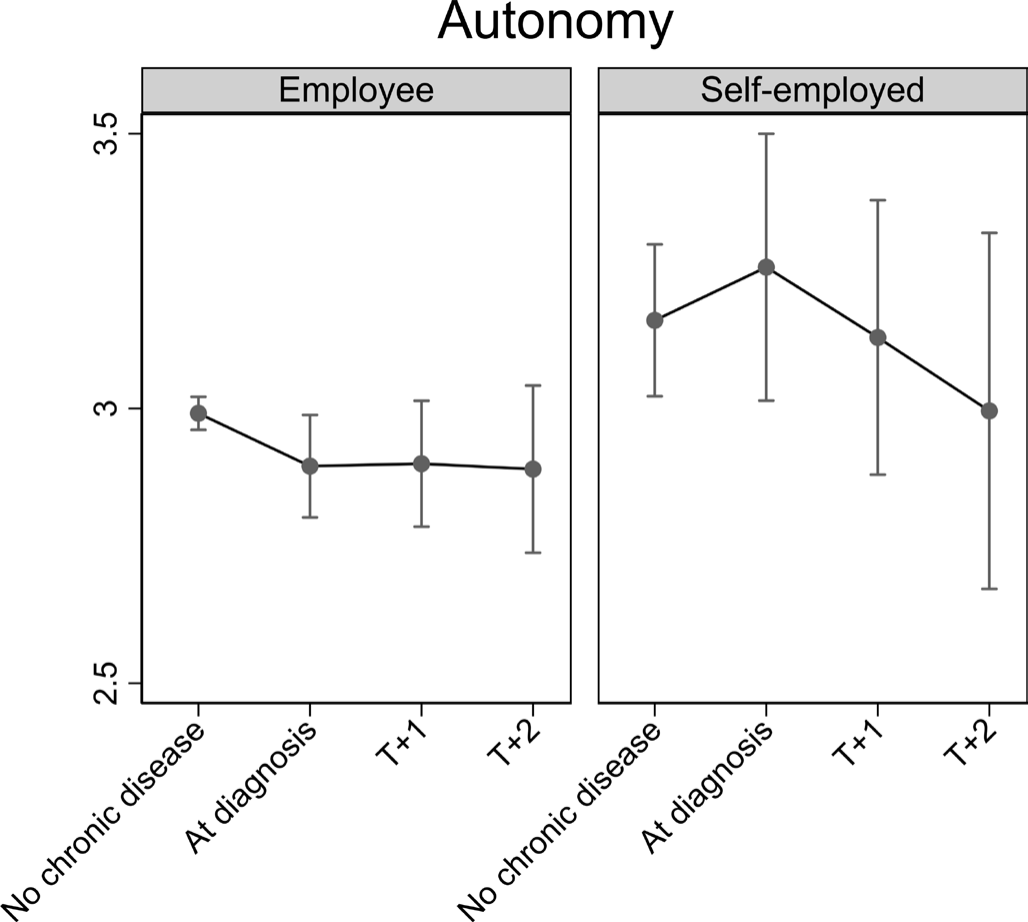
Impact of diagnosis of chronic disease on job autonomy for employed and self-employed older persons. Fully adjusted analyses.

**Figure 3:**
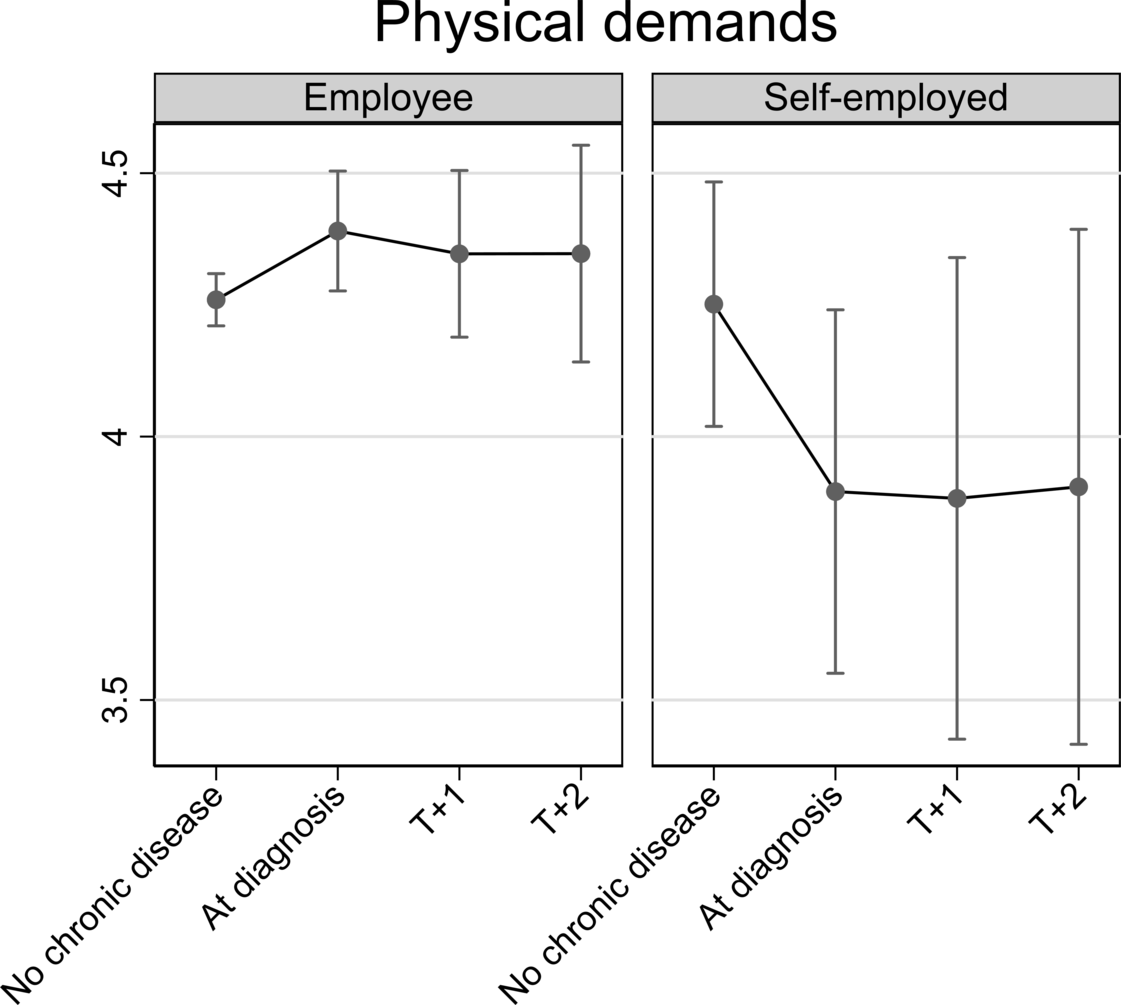
Impact of diagnosis of chronic disease on physical job demands for employed and self-employed older persons. Fully adjusted analyses.

**Figure 4:**
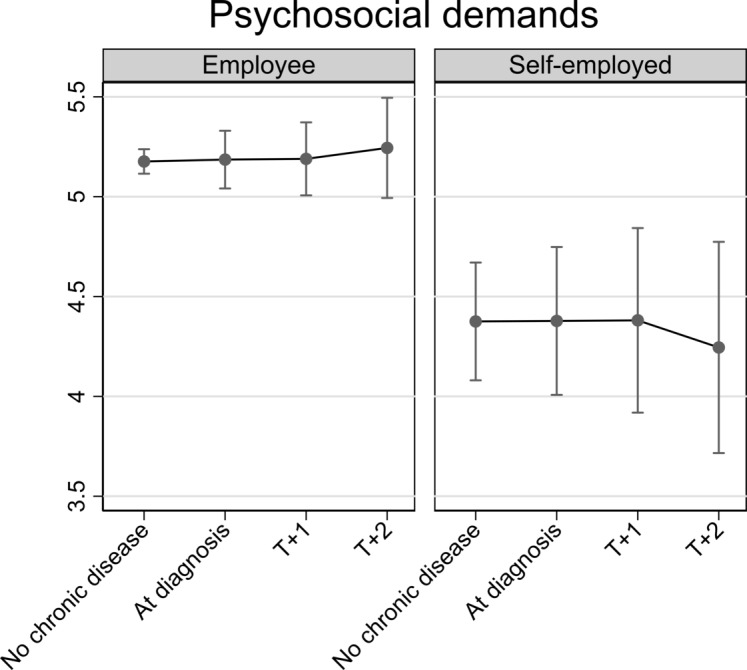
Impact of diagnosis of chronic disease on psychosocial job demands for employed and self-employed older persons. Fully adjusted analyses.

**Figure 5:**
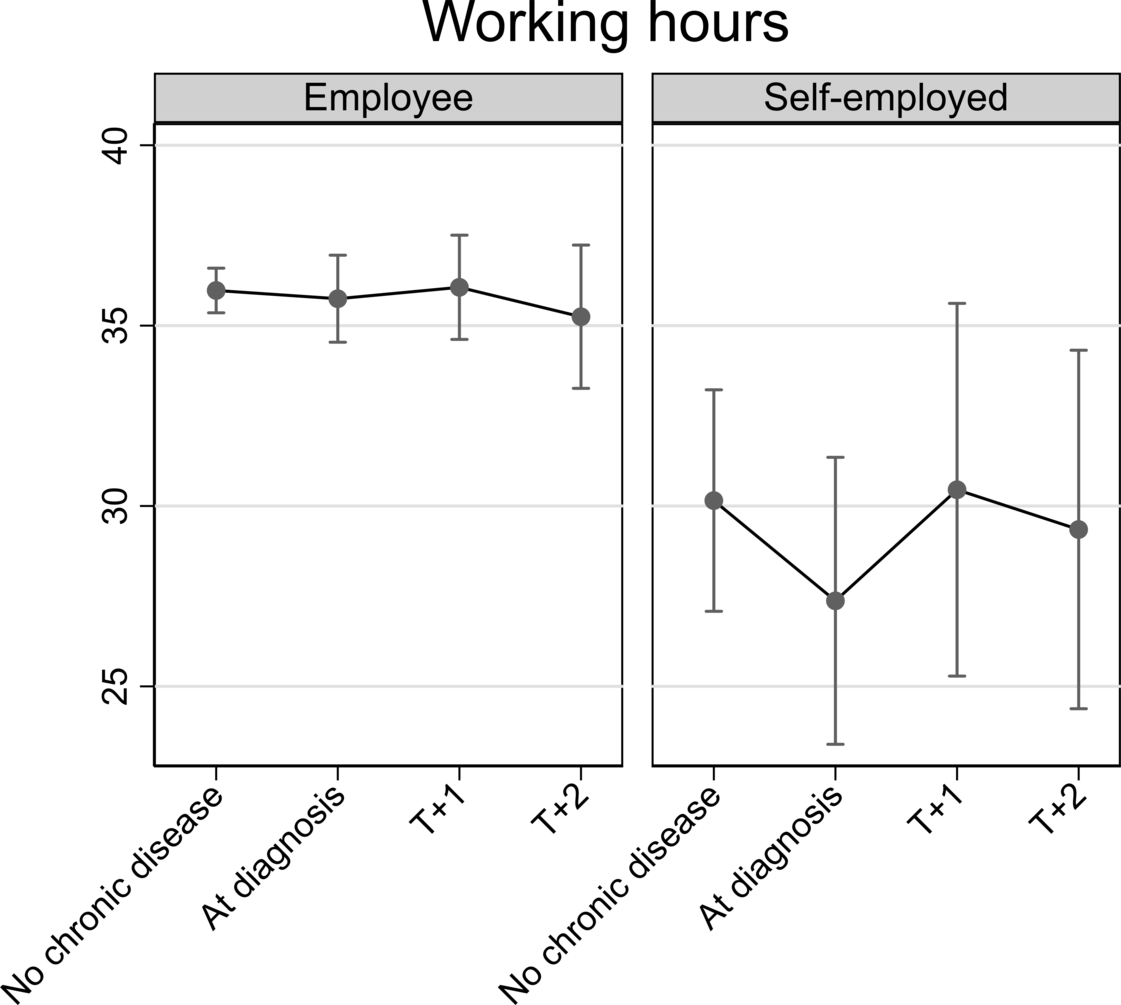
Impact of diagnosis of chronic disease on working hours for employed and self-employed older persons. Fully adjusted analyses.

All analyses were adjusted for categorical age, depression score, wave and job autonomy (latter only in models on job demands and working hours). The results from table 2 are depicted in figures 2–5, which respectively show changes in job autonomy, physical job demands, psychosocial job demands and working hours for employees and the self-employed, dependent on the incidence of diagnosis of chronic disease. Figures depict predicted scores, with covariates fixed at the sample mean. Before diagnosis of chronic disease, self-employed older persons report significantly higher job autonomy than employees (0.17, 95% CI 0.00 to 0.33). On diagnosis of chronic disease, employees reported decreased autonomy (−0.10, 95% CI −0.20 to 0.00). This decrease remains stable at one (T+1) and two waves (T+2) after diagnosis, but does not significantly differ from the prediagnosis level. For self-employed, we noted a slight increase in job autonomy on diagnosis of chronic disease, although this is not significantly higher compared with before diagnosis (0.10, 95% CI −0.13 to 0.32). This effect is not consistent at later time points after diagnosis of chronic disease. Physical job demands prior to diagnosis of chronic disease were on average similar for employees and self-employed persons. On diagnosis of chronic disease, employees’ physical job demands significantly increase (0.13, 95% CI 0.01 to 0.25), whereas the self-employed report significantly lower levels of physical job demands (−0.36, 95% CI −0.64 to –0.07), compared with levels of job demands before diagnosis. For both groups, this impact is also evident, one and two waves after diagnosis of chronic disease, but differences are no longer significant. Psychosocial job demands are on average significantly lower among the self-employed than employees before diagnosis of chronic disease (−0.63, 95% CI −0.98 to –0.28). Levels of psychosocial job demands for employees (0.01, 95% CI −0.14 to 0.16) or self-employed (0.00, 95% CI −0.30 to 0.31) did not significantly change on diagnosis. Prior to diagnosis of chronic disease, working hours among the self-employed are on average significantly lower than among employees (−5.15, 95% CI −8.82 to –1.48). On and following diagnosis of chronic disease, there is no significant change in employees’ working hours. For self-employed, we notice a drop in working hours on diagnosis of chronic disease (−2.78, 95% CI −6.03 to 0.48), although not statistically significant.

**Table 2:** Fixed-effects linear regression analysis investigating impact of diagnosis of chronic disease for working conditions

	(1) Autonomy†	(2) Physical demands‡	(3) Psychosocial demands‡	(4) Working hours‡
	Coef. (95% CI)	Coef. (95% CI)	Coef. (95% CI)	Coef. (95% CI)
No chronic disease	0	0	0	0
At diagnosis	−0.10+ (−0.20 to 0.00)	0.13*(0.01 to 0.25)	0.01(−0.14 to 0.16)	−0.23(−1.37 to 0.91)
T+1	−0.09 (−0.21 to 0.03)	0.09 (−0.08 to 0.25)	0.01 (−0.18 to 0.20)	0.09 (−1.37 to 1.54)
T+2	−0.10 (−0.26 to 0.06)	0.09 (−0.13 to 0.30)	0.07 (−0.19 to 0.33)	−0.73 (−2.74 to 1.29)
Self-employment	0.17* (0.00 to 0.33)	0.01 (−0.27 to 0.29)	−0.63*** (−0.98 to −0.28)	−5.15** (−8.82 to −1.48)
Moderation chronic disease # Self-employment				
No chronic disease # Self-employed	0	0	0	0
At diagnosis # Self-employed	0.19 (−0.05 to 0.44)	−0.49** (−0.79 to −0.18)	−0.01 (−0.34 to 0.33)	−2.55 (−5.98 to 0.88)
T+1 # Self-employed	0.06 (−0.21 to 0.33)	−0.46* (−0.89 to −0.02)	−0.01 (−0.46 to 0.44)	0.21 (−4.48 to 4.91)
T+2 # Self-employed	−0.06 (−0.42 to 0.29)	−0.43+ (−0.92 to 0.05)	−0.20 (−0.74 to 0.34)	−0.07 (−4.70 to 4.56)
Observations	4824	4824	4824	4824
Individual observations	1389	1389	1389	1389
Minimal number obs	2.00	2.00	2.00	2.00
Average number obs	3.5	3.5	3.5	3.5
Maximum number obs	6.00	6.00	6.00	6.00

* p<0.05, **p<0.01, ***p<0.001, +p<0.10.

†Model is adjusted for categorical age, CES-D depression score and wave.

‡Models are adjusted for categorical age, CES-D depression score, job autonomy and wave.

CES-D, Centre for Epidemiologic Studies Depression.

Online supplementary table 1–3 depict the sensitivity analyses, which can each best be compared with table 2. First, we restricted the analyses to those who were either self-employed or employed throughout (online supplementary table 1). We find that the onset of chronic disease affects employed older workers’ physical job demands similarly to the results presented previously in table 2. For the self-employed, however, the decrease in physical demands on diagnosis of chronic disease is less pronounced in the restricted analyses. It is noticeable in the sensitivity analyses that the self-employed report significantly higher physical job demands than employed older workers before diagnosis of chronic disease. On diagnosis of chronic disease, the difference in physical job demands between employed and self-employed older workers decreases. For autonomy, psychosocial job demands and working hours our results are similar to those reported in table 2.

10.1136/jech-2017-210328.supp1 Supplementary file 1

Second, onset of a second chronic disease was not associated with changes in working conditions, and adjustment of this did not materially affect the results (online supplementary table 2). Finally, when we accounted for an anticipation effect (online supplementary table 3), estimates of changes in working conditions for the self-employed and employees were consistent with analyses in table 2.

## Discussion and conclusion

Working conditions are known to be modifiable characteristics of work and can be adjusted when work capability changes, for example, due to chronic disease. We investigated whether older persons’ reported working conditions changed on diagnosis of chronic disease. We estimated this effect in relation to employment and self-employment because work modifications may be more feasible for the self-employed.^21 22^

We showed that employees reported significantly lower autonomy compared with the self-employed before diagnosis of chronic disease. On diagnosis, employees reported decreasing levels of autonomy, but this was only marginally significant and did not persist in later waves. Changes of autonomy in self-employed were not significant. Physical job demands slightly increased among employees on diagnosis of chronic disease, but decreased among the self-employed. This decline persisted postdiagnosis. Diagnosis of chronic disease did not affected psychosocial job demands among the self-employed or employees, but psychosocial job demands were significantly lower among self-employed, compared with employed older persons. With regard to working hours, the self-employed reduced their working hours on diagnosis of chronic disease, but no change was found among employees. The drop of working hours among self-employed was, however, not statistically significant and did not persist postdiagnosis. These results were fundamentally replicated in sensitivity analyses. Our results provide some support for the rationale that older workers with chronic disease are in need of work modifications.^19^ Given past studies showing that good working conditions can contribute to extending working lives,^4–6^ work modifications could help older workers to continue working. These modifications seem to be more feasible for the self-employed.

Our analyses showed that autonomy, psychosocial job demands and working hours differed for self-employed and employed older workers before diagnosis of chronic disease. On the one hand, this might be explained by the greater autonomy self-employed have in choosing their working conditions according to their individual preferences. On the other hand, this could point towards employment in different occupations, with different inherent working conditions. Additional analyses showed that self-employed, compared with employed, older workers were more frequently in intermediate occupations, rather than routine and manual occupations, but educational levels of the two groups were very comparable (results not shown, available on request).

This study benefits from a long follow-up period observing participants for up to six waves, covering about 12 years. In this period, individuals’ diagnosis of chronic disease and their changes in working conditions were observed. Individuals were observed up to 4 years (two waves) postdiagnosis. We employed fixed effects regression models, an advantageous method when investigating changes over time dependent on a treatment or event; in our study, the event is diagnosis of chronic disease. Unobserved heterogeneity from time-invariant confounders is accounted for in fixed-effects regressions, emphasising a potentially causal relationship. We set out to compare changes in working conditions of self-employed and employed older workers and provide insights into a group that has not been studied widely, even though an increasing number of older persons are in self-employment.

Our study has some limitations. First, our sample was restricted to persons aged 50–60 years at baseline, who were not yet diagnosed with chronic disease. This design implies that findings might not be representative of the older population before retirement, because chronic illness is a frequent condition in this age group. This design was, however, necessary to estimate an effect of onset of chronic disease, rather than comparing healthy and diseased older workers. Second, chronic disease was assessed by self-report rather than based on hospital records or reports by nurses or general practitioners. However, in ELSA, respondents were specifically asked about doctor’s diagnosis of the disease. Moreover, self-reported health conditions have been found to be consistent with those reported by physicians, especially for hypertension and diabetes.^36 37^ In this study, we assessed chronic illness by one single measure. Due to small sample size, it was not feasible to investigate chronic conditions separately. We acknowledge this possible limitation and encourage future research to study consequences of various chronic diseases separately. This would be particularly interesting because medication for some chronic diseases might be better tolerated than for others, or illnesses might differ in severity. Hence, adjustments of working conditions might not be equally relevant for all chronically ill workers. Another limitation is that we cannot rule out the possibility that individuals’ perception and/or reporting of working conditions changed in response to diagnosis of chronic disease. For example, the reported increase in physical job demands or decrease in autonomy among employees on diagnosis might solely be perceived, while objectively no change could have occurred. A perceived deterioration of working conditions might, however, indicate older persons’ higher stress from similar levels of demands after diagnosis of chronic disease, compared with before. It would, thus, still allow concluding that older persons might require adjustments in working conditions following diagnosis of chronic disease. Finally, older persons could also leave work as a response to unfeasible working conditions after diagnosis of chronic disease. By including respondents with at least two (and on average 3.5) observations in employment, we tried to include those with long follow-up.

Our study shows that changes in working conditions after diagnosis of chronic disease were more explicit among self-employed than employed older workers. In an ageing society, older persons are expected to work ever longer and potentially remain working despite chronic diseases, our findings should encourage employers, policy-makers and governments to consider and offer work modifications for chronically ill older adults.

**What is already known on this subject**

- Older persons with chronic disease may have different work requirements and lower work capability, compared with workers in good health.
- Adjusting working conditions may help older persons with chronic disease to remain in work.

**What this study adds**

- We show that a diagnosis of chronic disease is succeeded by a drop in physical job demands in the self-employed, but a slight increase in employees. No or little changes were found for self-employed or employees with regard to autonomy, psychosocial job demands and working hours.
- Work(place) adjustments for chronically ill workers could help prevent early exit from work.
