# Supplementary material for: Changes in autonomy, job demands and working hours after diagnosis of chronic disease: a comparison of employed and self-employed older persons using the English Longitudinal Study of Ageing (ELSA)
Source: J Epidemiol Community Health. 2018 Jun 23;72(10):951–7. doi: 10.1136/jech-2017-210328 (PMC6161656; doi:10.1136/jech-2017-210328)
Supplement: Supplementary file 1 [file jech-2017-210328supp001.pdf]

SUPPLEMENTARY MATERIAL

Table 1: Fixed effects linear regression analysis investigating impact of diagnosis of chronic disease for working conditions. Analyses restricted to respondents who were either self-employed throughout or employee throughout.

	(1) Autonomy[a] Coef. [95% CI]	(2) Physical demands[b] Coef. [95% CI]	(3) Psychosocial demands[b] Coef. [95% CI]	(4) Working hours[b] Coef. [95% CI]
No chronic disease	0	0	0	0
At diagnosis	-0.10+	0.16**	-0.00	-0.02
	[-0.21,0.01]	[0.04,0.28]	[-0.15,0.15]	[-1.11,1.06]
T+1	-0.09	0.13	-0.04	-0.16
	[-0.22,0.03]	[-0.04,0.29]	[-0.23,0.15]	[-1.64,1.31]
T+2	-0.11	0.11	0.06	-0.64
	[-0.27,0.06]	[-0.10,0.33]	[-0.21,0.34]	[-2.65,1.37]
Self-employment	0.46***	0.65***	0.55***	-3.28**
	[0.29,0.63]	[0.44,0.87]	[0.31,0.79]	[-5.30,-1.26]
Moderation chronic disease * Self-employment				
No chronic disease # Self-employed	0	0	0	0
At diagnosis # Self-employed	0.09	-0.39*	0.08	-1.05
	[-0.20,0.38]	[-0.75,-0.03]	[-0.29,0.45]	[-4.79,2.69]
T+1 # Self-employed	-0.09	-0.26	0.14	-0.74
	[-0.41,0.22]	[-0.74,0.22]	[-0.30,0.58]	[-4.50,3.01]
T+2 # Self-employed	-0.14	-0.21	-0.24	-2.10
	[-0.55,0.27]	[-0.73,0.32]	[-0.76,0.29]	[-6.38,2.19]
Observations	4,367	4,367	4,367	4,367
Individual observations	1,263	1,263	1,263	1,263
Minimal number obs	2.00	2.00	2.00	2.00
Average number obs	3.46	3.46	3.46	3.46
Maximum number obs	6.00	6.00	6.00	6.00

Note: *** p<.001, ** p<.01, * p<.05 + p<.10

[a] Model is adjusted for categorical age, CES-D depression score, wave

[b] Models are adjusted for categorical age, CES-D depression score, job autonomy, wave

Table 2: Fixed effects linear regression analysis investigating impact of diagnosis of chronic disease for working conditions. Analyses are additionally adjusted for second chronic disease.

	(1) Autonomy[a] Coef. [95% CI]	(2) Physical demands[b] Coef. [95% CI]	(3) Psychosocial demands[b] Coef. [95% CI]	(4) Working hours[b] Coef. [95% CI]
No chronic disease	0	0	0	0
At diagnosis	-0.09	0.13*	0.03	-0.31
	[-0.19,0.02]	[0.01,0.25]	[-0.12,0.18]	[-1.49,0.86]
T+1	-0.08	0.08	0.04	-0.04
	[-0.20,0.05]	[-0.09,0.25]	[-0.15,0.24]	[-1.57,1.49]
T+2	-0.08	0.08	0.11	-0.90
	[-0.24,0.08]	[-0.14,0.30]	[-0.15,0.37]	[-2.96,1.16]
Self-employment	0.16+	0.01	-0.63***	-5.15**
	[-0.00,0.33]	[-0.27,0.29]	[-0.98,-0.28]	[-8.82,-1.48]
Moderation chronic disease * Self-employment				
No chronic disease # Self-employed	0	0	0	0
At diagnosis # Self-employed	0.18	-0.48**	-0.03	-2.47
	[-0.06,0.43]	[-0.79,-0.18]	[-0.36,0.31]	[-5.92,0.98]
T+1 # Self-employed	0.05	-0.45*	-0.02	0.28
	[-0.22,0.32]	[-0.89,-0.02]	[-0.47,0.43]	[-4.44,5.00]
T+2 # Self-employed	-0.06	-0.43+	-0.20	-0.06
	[-0.42,0.29]	[-0.92,0.05]	[-0.74,0.33]	[-4.70,4.58]
2nd chronic disease (y/n)	-0.10	0.03	-0.18	0.78
	[-0.31,0.11]	[-0.25,0.31]	[-0.45,0.09]	[-1.57,3.13]
Observations	4,824	4,824	4,824	4,824
Individual observations	1,389	1,389	1,389	1,389
Minimal number obs	2.00	2.00	2.00	2.00
Average number obs	3.47	3.47	3.47	3.47
Maximum number obs	6.00	6.00	6.00	6.00

Note: *** p<.001, ** p<.01, * p<.05 + p<.10

[a] Model is adjusted for categorical age, CES-D depression score, wave

[b] Models are adjusted for categorical age, CES-D depression score, job autonomy, wave

Table 3: Fixed effects linear regression analysis investigating impact of diagnosis of chronic disease for working conditions. Analyses additionally include an anticipation effect.

	(1) Autonomy[a] Coef. [95% CI]	(2) Physical demands[b] Coef. [95% CI]	(3) Psychosocial demands[b] Coef. [95% CI]	(4) Working hours[b] Coef. [95% CI]
Anticipation effect	-0.01	-0.05	-0.05	-0.34
	[-0.11,0.09]	[-0.18,0.07]	[-0.20,0.10]	[-1.60,0.92]
No chronic disease	0	0	0	0
At diagnosis	-0.10+	0.10	-0.02	-0.44
	[-0.22,0.01]	[-0.05,0.24]	[-0.21,0.16]	[-1.97,1.08]
T+1	-0.10	0.05	-0.02	-0.15
	[-0.24,0.04]	[-0.14,0.24]	[-0.24,0.20]	[-1.95,1.65]
T+2	-0.11	0.04	0.03	-0.99
	[-0.29,0.07]	[-0.19,0.28]	[-0.26,0.32]	[-3.32,1.34]
Self-employment	0.17*	0.01	-0.63***	-5.16**
	[0.00,0.33]	[-0.27,0.28]	[-0.98,-0.28]	[-8.83,-1.49]
Moderation chronic disease * Self-employment				
No chronic disease # Self-employed	0	0	0	0
At diagnosis # Self-employed	0.19	-0.48**	-0.00	-2.51
	[-0.05,0.44]	[-0.78,-0.18]	[-0.34,0.33]	[-5.94,0.91]
T+1 # Self-employed	0.06	-0.45*	-0.00	0.25
	[-0.21,0.33]	[-0.89,-0.01]	[-0.45,0.45]	[-4.43,4.93]
T+2 # Self-employed	-0.06	-0.43+	-0.19	-0.04
	[-0.41,0.29]	[-0.91,0.05]	[-0.73,0.34]	[-4.67,4.59]
Observations	4,824	4,824	4,824	4,824
Individual observations	1,389	1,389	1,389	1,389
Minimal number obs	2.00	2.00	2.00	2.00
Average number obs	3.47	3.47	3.47	3.47
Maximum number obs	6.00	6.00	6.00	6.00

Note: *** p<.001, ** p<.01, * p<.05 + p<.10

[a] Model is adjusted for categorical age, CES-D depression score, wave

[b] Models are adjusted for categorical age, CES-D depression score, job autonomy, wave
